# Preventive Orthodontia*Read before the Alumni of the International School of Orthodontia, Kansas City, July 14, 1921. Reprinted from *International Journal*.

**Published:** 1921-11

**Authors:** C. M. McCauley


### PREVENTIVE ORTHODONTIA*

BY C. M. MCCAULEY, B.S., D.D.S.

In quite recent years the most familiar phrase among doctors was "remove the cause." Present day thought of the most advanced minds devoted to the healing art is toward prevention. Instead of waiting for a cause to produce a condition, and then treat the condition, the predominant idea is to discover the cause upon its appearance and remove it, thus preventing the condition. In preventive orthodontia one may go a step further. The etio-

* Read before the Alumni of the International School of Orthodontia, Kansas City, July 14, 1921. Reprinted from *International Journal.*

logic factors which combine to bring about what is looked upon as the direct cause of many cases of malocclusion may often be obliterated before the visible cause appears in sight. That it is better to prevent than to cure, no one will question.

There are two outstanding reasons why preventive orthodontia should appeal most strongly to the consideration of all persons interested in prevention of general diseases:

1. Because of the vast importance to the general health and appearance of the individual. A large percentage of nose, throat, and sinus trouble would never occur. Caries of temporary teeth would be greatly diminished. Permanent teeth would be more immune to disease and mastication would be better performed. Better digestion and assimilation, and all the good things which result therefrom, would be ours. Pyorrhea of later years would be greatly diminshed. Alveolar abscess and all its dreadful sequelæ would be lessened. The opsonic index would be kept higher and many childhood diseases of extra-oral origin would be avoided.

2. Because we know what the etiologic factors are in a majority of cases. They are of such a nature that we may see them before they actually become visible to the eye. They can always be detected when they appear and in many cases before. Yes, these causative factors may be known to the close observer oftentimes several years before the actual deformity appears. All of which makes it less excusable to leave them unobserved while Nature has tried in the face of too many difficulties to produce a good denture and failed.

Preventive orthodontia may be considered the basis of all prevention because it seeks to build perfect dentures, and perfect dentures are necessary to perfect digestion and assimilation. Besides that, when basic prevention is truly accomplished and a perfect denture completed, the mouth as a center of focal infection will be practically eliminated, and the nose and throat as factors in focal infection will be greatly reduced. The growth and development of the mouth and its tissues are so closely associated with the growth and development of the nasal passages and their tissues that the obstructions to normal processes originating in either organ will cause trouble in the other.

### MORE MODERN CONCEPTION OF ORTHODONTIA.

This specialty, in the earlier years of its existence, sought only to straighten crooked teeth and place them in alignment. In later years it embraced the science of occlusion. Advanced orthodontia of the present day covers not only the correct alignment of teeth in their arches, in a manner known as normal occlusion, but it seeks also to establish normal proportions in facial outline and to produce harmony in all facial features which owe their origin to dentofacial relations.

The orthodontist who has a true vision of the scope of his work, who sees the many factors in its etiology and appreciates the benefits to his patient made possible by inhibiting the action of or removing these factors, cannot avoid a deep feeling of responsibility to those who submit their cases to his judgment. Preventive orthodontia has many phases and will cover a larger field of work than that formerly done by orthodontists but its reward will also be greater. The patient who receives proper attention in this line of prevention will build an adequate foundation upon which the whole structure of health will rest, and it is difficult to conceive the great number of blessings resulting.

Normal development and prevention in orthodontia work side by side. Natural processes preside over normal development and when these processes fail it is the duty of the orthodontist to come to the rescue immediately when this failure occurs in tooth development, and render assistance. Such failures on the part of Nature in building the denture are due to pathological conditions. It therefore becomes the duty of the orthodontist to know or to learn when normal growth ceases and pathologic processes commence. In order to so differentiate he must be able to recognize the normal as well as the abnormal.

### NORMAL DEVELOPMENT

Normal development of the denture depends primarily upon proper development of the individual. Also the proper functioning of the denture has great bearing upon general development. Primarily the development of the individual depends upon nutrition, environment and inheritance. Normal development is normal growth plus the organization of tissues to meet the demand of special function. Such development would be the result in all cases were it not for antagonizing forces from disease or malnutrition which interfere with normal denture building. This interference becomes stronger in its efforts to abort the processes of growth than are those forces of Nature which are trying to produce a perfect denture. When this happens, distortion and maldevelopment are the result. The purpose of preventive orthodontia is to recognize these antagonizing forces as soon as they appear and by artificial means supply the necessary stimulus to assist Nature in overcoming them.

### SOME CONDITIONS ACCOMPANYING NORMAL DEVELOPMENT OF TEETH

Some of the physical forces which direct the teeth into correctly formed arches are the following:

1. The tongue and muscles of deglutition which influence the shape and curvature of the arches by outward pressure.

2. The muscles and cheeks which exert a pressure in the opposite direction from that of the tongue, thus establishing an area of equilibrium into which the teeth are at liberty to go.

3. The mouth closed and lips together, the lower lip overlapping the maxillary anterior teeth.

4. Well ventilated nasal passages for free passages of air.

5. Growth of the teeth and surrounding tissues in occlusal, forward and outward directions.

6. Long cusps and inclined planes of deciduous teeth which will develop the arches laterally if vigorous use is made of the teeth during mastication.

From the foregoing we may deduce the following along preventive lines:

1. Proper diet to influence normal growth. This applies not only to the child after birth, but to the mother before birth, and while nursing the child. The percentage of bottle-fed babies who develop malocclusion is considerably larger than that of babies fed on human milk. Many cases of malocclusion due to malnutrition may be prevented by correct diet.

2. If deficiency in size of the jaw bone has resulted from errors in development, there will not be sufficient space for normal eruption of permanent teeth, and the result will be a crowded and irregular denture. When the permanent anterior teeth erupt normally, they appear in the arch in their right positions. About two years before eruption their growth begins to cause enlargement of the dental arches as shown by the spaces which appear between the anterior deciduous teeth. This enlargement is facilitated by the permanent teeth maintaining their right positions, with their longest diameters in the line of the arch. In other words if they do not maintain their right positions before and during eruption and stimulate the growth of bone as they should, some additional force is necessary. If the spacing fails to appear between the deciduous teeth at the proper time, namely between the fourth and sixth year, the case should be examined with the X-ray and if the permanent teeth are found to be crowded and irregular, treatment should be started at once. At this stage, treatment is usually more simple and easy than at any future time. Only slight pressure upon the lingual surfaces sufficient to stimulate the tissues slightly will cause the arches to widen and provide room for permanent teeth to erupt. This force may be applied with a small lingual bar or with any device which will apply the force in the right way. Oftentimes the maxillary arch will not require any attention as the expansion of the mandibular will expand the maxillary by means of the cuspal inclined planes in occlusion. It is possible to prevent serious cases of neutroclusion by this simple procedure if the case is discovered in time.

3. Hyperplasia of lymphoid tissue in the nasopharynx which closes the nasal passages and forces the mouth open for breathing, lays the foundation at a very early age for malocclusion. These patients should have attention as early as possible, not later, however, than the fourth year. Proper treatment of such conditions administered early enough would prevent much trouble in later years.

4. All malocclusion resulting from loss of deciduous teeth from caries could be prevented if the child had proper attention at the right time. Caries may occur at any time after the eruption of the deciduous teeth. Therefore the age of prevention would begin at the time of eruption of these teeth. Surely four years of age is not too early to begin efforts to prevent caries. There is too great a tendency among the laity to neglect the first teeth of the children because they are regarded of little value. They are only deciduous according to their view point and will be lost in a few years any way. No need to have one filled because the filling will also be lost when the tooth goes. As a matter of fact, when the health of the child is considered, and the deciduous teeth are valued at their real worth as a factor in the foundation for future health of the individual, the deciduous denture is worth as much if not more than the permanent. Not only should each tooth be retained in its place until the proper time for its shedding, but each contact point should be maintained with utmost care. The matter of keeping the temporary denture intact and perfect in its function is one of the most important phases of preventive orthodontia.

We know the cause of a great many cases of malocclusion. There is no question about the cause in a multitude of cases. These causes can be removed or prevented if we only get the cases in time and handle them intelligently. If our literature contained more about ways and means of securing our patients earlier, about detecting deformities before they come into view from eruption of the second teeth and suggesting methods of preventive procedure at the right time, it would tend toward the elevation of the already high standard of the specialty. We take up too much space discussing appliances in our literature. Of course we must have appliances, but there is too much said about them to the exclusion of other things of importance. My experience with appliances has taught me that teeth are moved by a combination of appliance and personal element in the operator. The difference in the personal element tells us why one man gets results with one appliance and another man gets equally good results with an entirely different appliance. Each operator should use the appliance which, in combination with the personal element within him, secures the best results with the case in hand. Different movements of teeth require different methods of applying force, which fact makes it wise to use a different appliance when indicated. In the judgment of the writer it would be a good thing for our national society, or societies, to devote a meeting to discussions of preventive procedure, outlining the scope of the orthodontist's work as regards prevention. It is quite probable that such a meeting would change the character of our programs for the future.

### CONCLUSION

A very large percentage of cases of malocclusion is the result of interference with Nature's processes of development at a time when orthodontia could render the assistance necessary to prevent the disorder. To do this successfully we should have our patients at a younger age than we are getting them now. Suppose we make an effort to secure the child for examination before the fourth birthday, and if an abnormality is seen correct it or see that it is corrected, then watch the case at frequent and regular intervals thereafter until the permanent denture is correctly completed. If there is caries have it arrested. If there is loss of normal contact in temporary teeth have it restored. If prophylaxis is needed, see that it is provided. If movement of teeth is needed, do it now.

